# Transiently proliferating perivascular microglia harbor M1 type and precede cerebrovascular changes in a chronic hypertension model

**DOI:** 10.1186/s12974-019-1467-7

**Published:** 2019-04-10

**Authors:** Takashi Koizumi, Katsutoshi Taguchi, Ikuko Mizuta, Hiroe Toba, Makoto Ohigashi, Okihiro Onishi, Kazuya Ikoma, Seiji Miyata, Tetsuo Nakata, Masaki Tanaka, Sébastien Foulquier, Harry W. M. Steinbusch, Toshiki Mizuno

**Affiliations:** 1 Department of Neurology, Graduate School of Medical Science, Kyoto Prefectural University of Medicine, 465 Kajii-cho Kamigyo-ku, Kyoto, 602-8566 Japan; 2 Department of Anatomy and Neurobiology, Graduate School of Medical Science, Kyoto Prefectural University of Medicine, Kyoto, Japan; 3 Department of Clinical Pharmacology, Division of Pathological Sciences, Kyoto Pharmaceutical University, Kyoto, Japan; 4 Department of Orthopaedics, Graduate School of Medical Science, Kyoto Prefectural University of Medicine, Kyoto, Japan; 5 Department of Applied Biology, Kyoto Institute of Technology, Kyoto, Japan; 6 Department of Pharmacology and Toxicology, School for Mental Health and Neuroscience, Maastricht University, Maastricht, the Netherlands; 7 Department of Neuroscience, School for Mental Health and Neuroscience, Maastricht University Medical Center +, Maastricht, The Netherlands

**Keywords:** Neuroinflammation, Cerebral small vessel disease, Chronic hypertension, Perivascular microglia, Proliferation

## Abstract

**Background:**

Microglia play crucial roles in the maintenance of brain homeostasis. Activated microglia show a biphasic influence, promoting beneficial repair and causing harmful damage via M2 and M1 microglia, respectively. It is well-known that microglia are initially activated to the M2 state and subsequently switch to the M1 state, called M2-to-M1 class switching in acute ischemic models. However, the activation process of microglia in chronic and sporadic hypertension remains poorly understood. We aimed to clarify the process using a chronic hypertension model, the deoxycorticosterone acetate (DOCA)-salt-treated Wistar rats.

**Methods:**

After unilateral nephrectomy, the rats were randomly divided into DOCA-salt, placebo, and control groups. DOCA-salt rats received a weekly subcutaneous injection of DOCA (40 mg/kg) and were continuously provided with 1% NaCl in drinking water. Placebo rats received a weekly subcutaneous injection of vehicle and were provided with tap water. Control rats received no administration of DOCA or NaCl. To investigate the temporal expression profiles of M1- and M2-specific markers for microglia, the animals were subjected to the immunohistochemical and biochemical studies after 2, 3, or 4 weeks DOCA-salt treatment.

**Results:**

Hypertension occurred after 2 weeks of DOCA and salt administration, when round-shaped microglia with slightly shortened processes were observed juxtaposed to the vessels, although the histopathological findings were normal. After 3 weeks of DOCA and salt administration, M1-state perivascular and parenchyma microglia significantly increased, when local histopathological findings began to be observed but cerebrovascular destruction did not occur. On the other hand, M2-state microglia were never observed around the vessels at this period. Interestingly, prior to M1 activation, about 55% of perivascular microglia transiently expressed Ki-67, one of the cell proliferation markers.

**Conclusions:**

We concluded that the resting perivascular microglia directly switched to the pro-inflammatory M1 state via a transient proliferative state in DOCA-salt rats. Our results suggest that the activation machinery of microglia in chronic hypertension differs from acute ischemic models. Proliferative microglia are possible initial key players in the development of hypertension-induced cerebral vessel damage. Fine-tuning of microglia proliferation and activation could constitute an innovative therapeutic strategy to prevent its development.

**Electronic supplementary material:**

The online version of this article (10.1186/s12974-019-1467-7) contains supplementary material, which is available to authorized users.

## Background

Microglia are the resident immune cells in the brain and play pivotal roles in environmental surveillance to maintain brain homeostasis. Inflammation or cellular damage can stimulate microglia to increase the activity of immune functions [1]. In vivo two-photon microscopy studies showed that activated microglia rapidly migrate to and accumulate at sites of pathological lesions, such as ischemic lesions [2] or newly formed amyloid-β plaques [3].

The activated microglia show a biphasic influence, promoting beneficial repair and causing harmful damage. Those responsible for the former are sometimes referred to as anti-inflammatory M2 microglia and the latter as pro-inflammatory M1 microglia [4]. These different types of activated microglia can be distinguished based on the expression of specific markers. These activated microglia are involved in various neurological disorders [1, 5] and also influence the function and integrity of the blood-brain barrier (BBB) [6, 7]. In the present study, we focused on the interaction between microglia dynamics and cerebrovascular disease.

According to previous studies, microglia are activated in the acute phase of ischemic stroke as shown in animal models of transient middle cerebral artery occlusion (tMCAO) [8–10]. In the tMCAO model, numbers of M2-state microglia rapidly increase around vessels in the penumbra after infarction, and, in a few days, M1-state microglia dominantly increase. This process is called “M2-to-M1 phenotype-switching” or “shift in the M2-to-M1 phenotypes” [11, 12]. In addition to microglia activation, various molecules are involved in the acute phase of ischemic strokes, such as free radicals [13], damage-associated molecular patterns (DAMPs) [14], and T cells [15]. DAMPs include heat shock protein, high-mobility group box 1 (HMGB1), and peroxiredoxin [14]. For example, HMGB1 is produced by ischemic neuronal cells about 2–4 h after an ischemic event and peaks at around 6 h. HMGB1 affects vascular endothelial cells and induces BBB destruction, as well as microglia activation [16].

In contrast, in cerebral small vessel disease (CSVD), only a limited number of reports referred to M1 and M2 microglia phenotyping and their molecular mechanisms. Chronic hypertension being the major risk factor of CSVD [17, 18], we focused on a hypertensive CSVD model.

The aim of this study is to clarify microglia involvement in CSVD caused by chronic hypertension using deoxycorticosterone acetate (DOCA)-salt-treated Wistar rat (DOCA-salt rat) as a model mimicking sporadic and chronic hypertension.

## Methods

### Animals

Adult male Wistar rats (150–180 g) were purchased from SHIMIZU Laboratory Supplies Co, Ltd. (Kyoto, Japan). Protocols were approved by Animal Care and Use Committees of Kyoto Prefectural University of Medicine and Kyoto Pharmaceutical University. We studied Wistar rats fed standard chow and water ad libitum. Care and use of rodents met the standards set by the National Institutes of Health for experimental animals. The rats were housed under specific pathogen-free conditions and fed standard laboratory chow and water ad libitum before entering the study. They were maintained on a 12-h light/day cycle at 20–22 °C and 40–50% humidity.

### Preparation of the chronic hypertension model

**Fig. 1:**
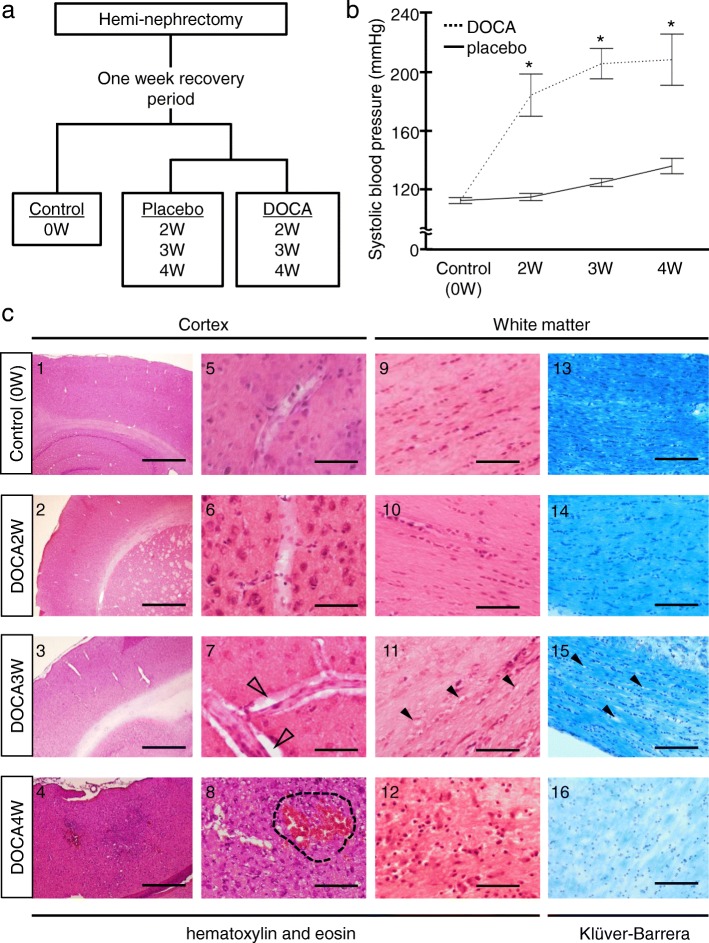
Progression of hypertension and histological damage in deoxycorticosterone acetate (DOCA)-salt rats. **a** Experimental grouping for histological analysis of the model animals. **b** Systolic blood pressure in DOCA2W, DOCA3W, and DOCA4W was compared with that in the control. Values are expressed as the means ± SEM (*n* = 3 in each group, *p < 0.05). **c** Hematoxylin and eosin or Klüver-Barrera staining of brain tissues. In DOCA3W, focal vascular remodeling including the perivascular space enlargements (open arrowheads) and the formation of vacuoles in the white matter (closed arrowheads) appeared. In DOCA4W, cerebral hemorrhage (dotted area) and myelinoclasis lesions were found. Scale bars 500 μm (1–4), 20 μm (5–12), and 40 μm (13–16)

Rats were anesthetized by combination anesthesia administered i.p. with 0.375 mg/kg of medetomidine, 2.0 mg/kg of midazolam, and 2.5 mg/kg of butorphanol and underwent unilateral nephrectomy. After a recovery period of 7 days, the rats were randomly divided into a deoxycorticosterone acetate (DOCA)-salt group, placebo group, and control (Fig. 1a). DOCA-salt group rats received a weekly subcutaneous injection of DOCA (40mg/kg body weight (Nacalai Tesque, Kyoto, Japan)) suspended in carboxymethylcellulose and were provided with 1% NaCl in drinking water for 2, 3, or 4 weeks (DOCA2W, DOCA3W, and DOCA4W, respectively, *n* = 3 for each). Placebo group rats received a weekly subcutaneous injection of vehicle and were provided with tap water for 2, 3, or 4 weeks (*n* = 3 for each period). Control rats (*n* = 3) received no administration of DOCA or NaCl. The rat was placed in a restraint cage in a warm (38 °C) condition for approximately 2–3 min, then systolic blood pressure and heart rate in a conscious state were measured by the tail-cuff method (BP-98A-L, Softron, Tokyo, Japan). The values were measured three times for each rat and the average value was calculated. After resting overnight under a light shield, the rats underwent magnetic resonance imaging (MRI) under anesthesia with the inhalation of isoflurane. Immediately after MRI, the rats were perfused transcardially with 4% paraformaldehyde in phosphate buffer, and their brains were removed. For immuno- or pathological staining, brains were post-fixed in the same fixative overnight at 4 °C and further cryoprotected sequentially in 5, 10, 15, and 25% sucrose. Brains embedded in optimal cutting temperature compounds were stored at − 20 °C until examination. Frozen sections of a brain were cut into 20-μm-thick slices with a cryostat (CM1850, Leica, Germany).

For biochemical analysis, fresh brain tissues rapidly frozen in liquid nitrogen were prepared from the control, DOCA2W, and DOCA3W (*n* = 4, in each group) rats under anesthesia.

### Evaluation items

**Fig. 2:**
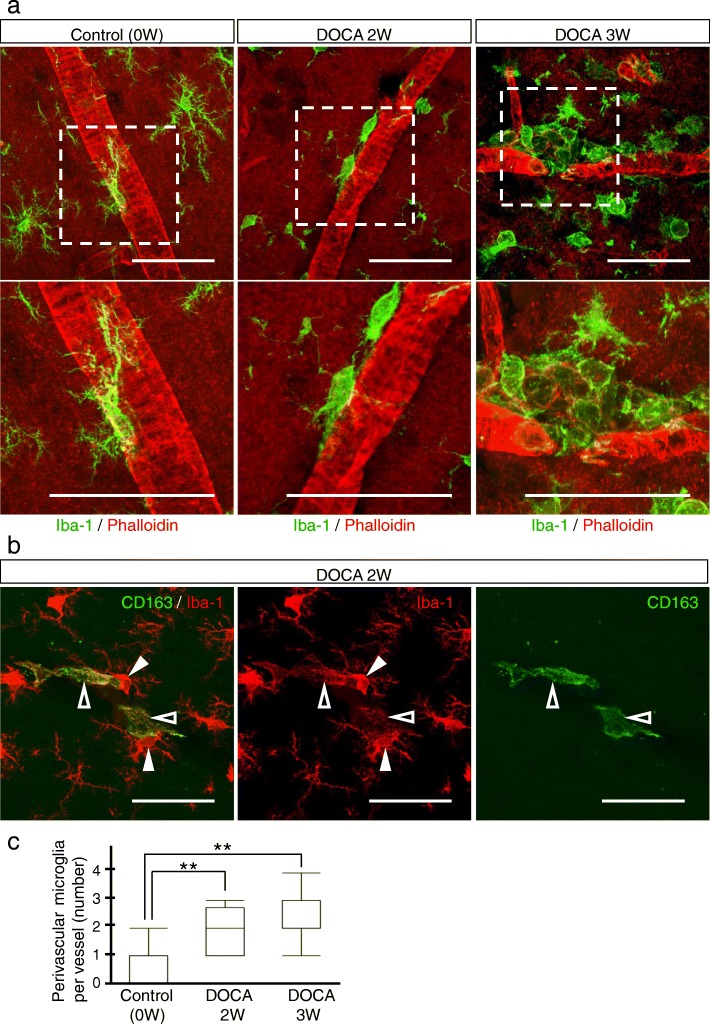
Dynamic morphological changes of microglia in DOCA-salt rats. **a** Microglia and the cerebral vasculature were visualized by Iba-1 (green) and phalloidin (actin, red), respectively, in the control (left column), DOCA2W (middle column), and DOCA3W (right column). The white dotted square in the upper row was magnified in the lower row. **b** Perivascular macrophages (CD163-positive cells, green) showed a flattened shape and low expression of Iba-1 (open arrowheads). In contrast, microglia had fine processes and showed high expression of Iba-1 (closed arrowheads). **c** Perivascular microglia increased in number after DOCA and salt treatments. Significance is expressed as **p < 0.01 using Tukey-Kramer test. Scale bars 50 μm

**Fig. 3:**
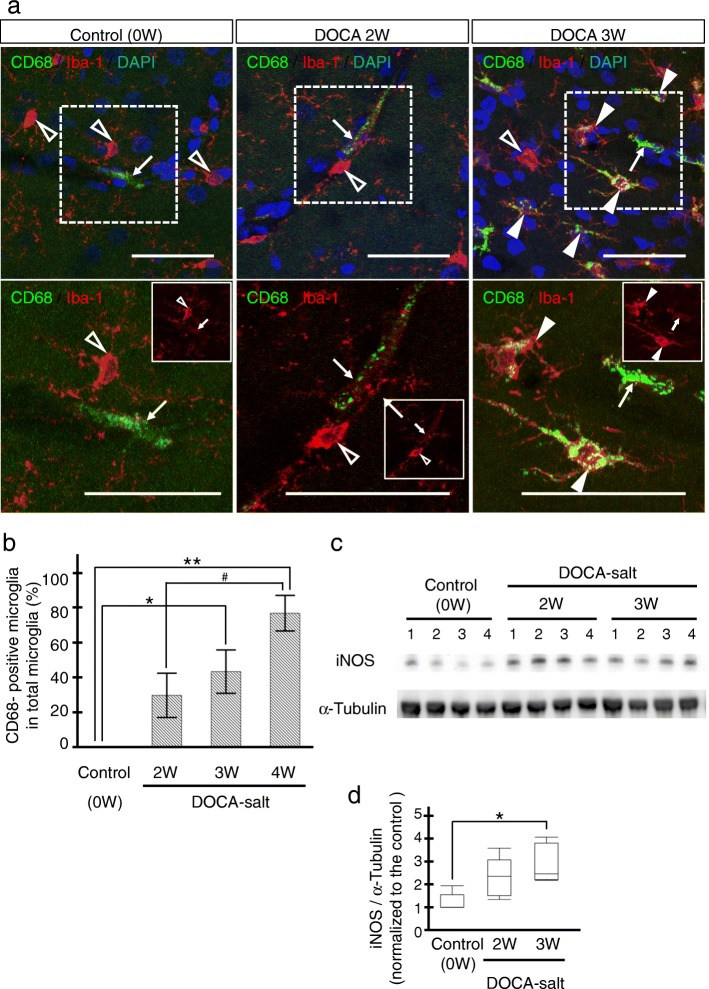
M1 switching of microglia in DOCA3W-rats. **a** Microglia and macrophages were visualized by Iba-1 (red) and CD68 (M1 marker, green), respectively, in the control (left column), DOCA2W (middle column), and DOCA3W (right column). The white dotted square in the upper row is magnified in the lower row. CD68-positive microglia were observed in DOCA3W (closed arrowheads) but not in DOCA2W (open arrowheads). In the white square, only Iba-1 staining was noted. Through the periods, CD68-positive perivascular macrophages existed (arrows). **b** CD68-positive microglia increased in number in DOCA3W. **c**, **d** The expression level of iNOS protein (M1 microglia marker) was increased after DOCA and salt treatment. Significant differences are expressed as *p < 0.05 and **p < 0.01 relative to the control rats and ^#^*P* < 0.05 relative to DOCA2W using Tukey-Kramer test. The values represent the means ± SEM. Scale bars 50 μm

**Fig. 4:**
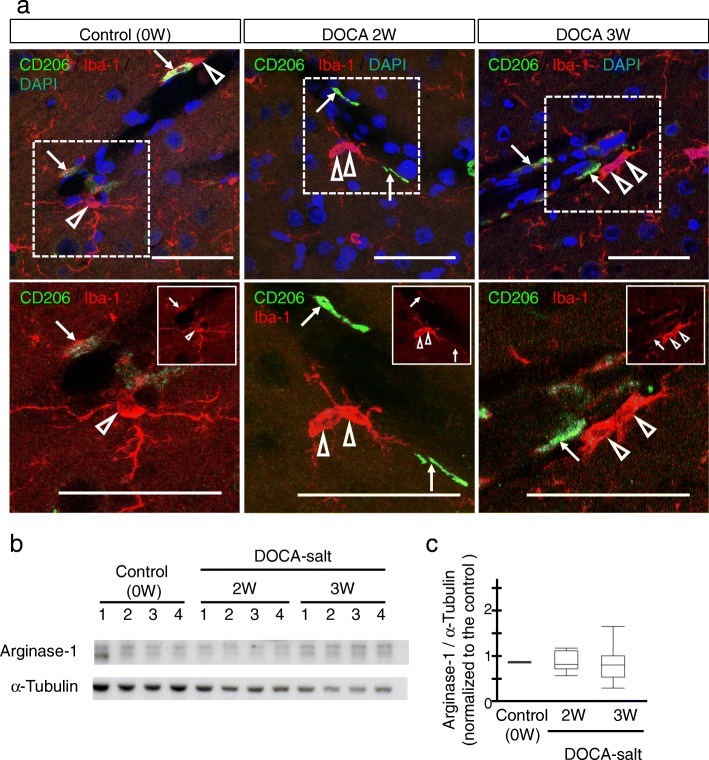
Absence of M2 switching in DOCA-salt rats. **a** Microglia and macrophages were visualized by Iba-1 (red) and CD206 (M2 marker, green) in the control (left column), DOCA2W (middle column), and DOCA3W (right column). The white dotted square in the upper row is magnified in the lower row. CD206-positive microglia were not observed in DOCA-salt rats (open arrowheads). In the white square, only Iba-1 staining was noted. CD206-positive perivascular macrophages were present in all periods (arrows). **b**, **c** No significant increase in the expression level of arginase-1 protein was observed after DOCA and salt treatment. Scale bars 50 μm

**Fig. 5:**
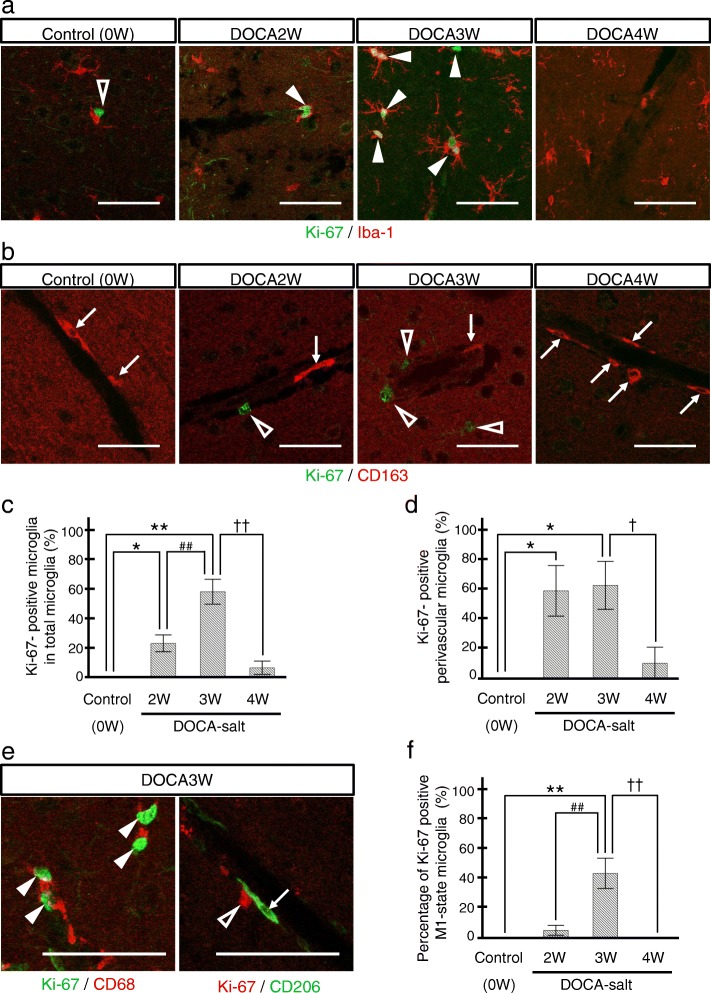
Transient increase of proliferative microglia before M1 switching. **a** Microglia were visualized by Iba-1 (red) and proliferative cells by Ki-67 (green). Ki-67-positive microglia were increased in DOCA2W and more markedly increased in DOCA3W (closed arrowheads). In DOCA4W, Ki-67-positive microglia were markedly decreased. **b** CD163-positive perivascular macrophages (red, arrows) were not merged with Ki-67 (green, open white arrowheads) in all stages. **c** The rate of Ki-67-positive microglia started to increase in DOCA2W. **d** The rate of Ki-67-positive perivascular microglia became significantly higher in DOCA2W. **e** In some microglia of DOCA3W, Ki-67 was co-expressed with CD68 but not with CD206. **f** The rate of proliferative M1-state microglia was significantly higher in DOCA3W. Significant differences are expressed as *p < 0.05 and **p < 0.01 relative to control rats, ^##^*p* < 0.01 relative to DOCA2W, and ^†^*p* < 0.05 and ^††^*p* < 0.01 relative to DOCA3W using Tukey-Kramer test. The values represent the means ± SEM. Scale bars 50 μm

We evaluated time-course changes in the blood pressure (Fig. 1b), clinical features (Table 1), histopathology (Fig. 1c and Additional file 1: Figure S2 and S5c), glial fibrillary acidic protein (GFAP) immunostaining of astrocyte foot processes as the basic framework of BBB (Additional file 1: Figure S1), and magnetic resonance imaging (MRI) (Additional file 1: Figure S5a and S5b) of the rats. Histopathological analysis was performed by hematoxylin and eosin (HE) and Klüver-Barrera (KB) staining. To examine the morphological dynamics of microglia in DOCA-salt rats, we visualized microglia by ionized calcium-binding adapter molecule 1 (Iba-1) (Figs. 2, 3, 4, and 5), pro-inflammatory M1-state microglia by CD68 (Fig. 3), anti-inflammatory M2-state microglia by CD206 (Fig. 4), and proliferative ability of microglia by Ki-67 (Fig. 5). We identified the vasculature by phalloidin or 4′, 6-diamino-2-phenylindole (DAPI) staining.

**Table 1:** Physical profiles of control, placebo-group, and DOCA-salt group rats

Group		Body weight (g)	Systolic blood pressure (mmHg)	Heart rate (/min)	Symptoms
Control	0W	165 ± 4.3	113.0 ± 2.8	427.3 ± 38	None
Placebo	2W	261 ± 3.9*	116.3 ± 3.4	414.3 ± 31	None
	3W	295 ± 16*	125.0 ± 3.7	412.3 ± 33	None
	4W	330 ± 11*	130.0 ± 7.3	413.0 ± 12	None
DOCA	2W	250.3 ± 8.3*	183.3 ± 24*	416.0 ± 50	None
	3W	253.0 ± 30*	204.3 ± 18*	407.3 ± 9.5	Hemiparesis, bleeding from the tail vein, and loss of appetite
	4 W	225.0 ± 14*	207.0 ± 30*	450.0 ± 29	Severe inactive state

Rats administrated with DOCA-salt or placebo for 2 weeks (DOCA2W or Placebo2W), 3 weeks (DOCA3W or Placebo3W), and 4 weeks (DOCA4W or Placebo4W) were analyzed. Values are expressed as means ± SEM (*n* = 3 in each group). Statistical significance was expressed as *p < 0.05 relative to the control by using *t* test

### Antibodies

Primary antibodies were used against Iba-1 (mouse monoclonal IgG, 1:500 (Millipore) or rabbit polyclonal IgG, 1:1000 (Wako)), inducible nitric oxide synthase (iNOS) (rabbit polyclonal IgG, 1:200 (Abcam)), CD68 (mouse monoclonal IgG, 1:500 (Bio-Rad)), Arginase-1 (rabbit polyclonal IgG, 1:2000 (Gene Tex)), CD206 (goat polyclonal IgG, 1:400 (R &D systems)), Ki-67 (rabbit polyclonal IgG, 1:2000 (Novocas)), and GFAP (mouse monoclonal IgG, 1:10000 (Millipore)). For detection of the primary antibodies, Alexa488 or Alexa594-conjugated secondary antibodies (anti-rabbit or mouse IgG, 1:2000 (Thermo Fisher)) were used. Vessel walls were visualized by Alexa594-conjugated phalloidin (1:500, (Thermo Fisher)).

### Immunohistochemistry

Free-floating sections were permeabilized with phosphate-buffered saline containing 0.1% Tween 20 (PBST) for 30 min at room temperature, and then, antigen retrieval was performed with citrate buffer for 20 min at 75 °C or for 15 min at 95 °C (anti-Ki-67 antibody). After blocking with 5% normal goat serum diluted in PBST overnight at 4 °C, the sections were incubated with primary antibody diluted with PBST for 2 days at 4 °C. For CD206 staining, donkey serum was used for blocking. Following washing with PBST, sections were incubated with appropriate secondary antibodies with DAPI for 2 h at room temperature. After PBS washing, the sections were mounted on slides with FluorSave Reagent (Merck Millipore, Darmstadt, Germany). For confocal observation, images were acquired as Z stacks (10–20 z sections, 1 μm apart, 1024 × 1024 pixels) using a Plan-Apochromat 63×/1.40 Oil DIC objective (Carl Zeiss, Oberkochen, Germany) with an inverted laser-scanning confocal microscope, LSM510 META (Carl Zeiss). Image analysis was performed using Zeiss LSM Image Browser. The image acquisition region including the cortical blood vessels was randomly selected. Ten images per rat were acquired. We identified the vasculature by phalloidin or DAPI staining. Microglia were distinguished from macrophages by the Iba-1 expression level and their morphology. The numbers of cells were manually counted in each image.

### Western blotting

Frozen whole-brain tissue was minced and homogenized with a Polytron homogenizer in ice-cold PBS (five times the brain weight) containing phenylmethylsulfonyl fluoride. The samples were then sonicated. Before Western blot analysis, protein concentrations were determined using the Lowry method. We adjusted the protein concentration to 2 mg/mL and loaded 10 μg of the total protein in each well. Proteins were separated by NuPAGE Bis-Tris 10% gels (Thermo Fisher) and transferred onto PVDF membranes (Millipore). The membranes were blocked with Blocking One (Nacalai) for 1 h, followed by overnight incubation at 4 °C with primary antibodies in the blocking solution. Primary antibodies for iNOS (mouse monoclonal IgG (Pharmingen)), Arginase-1 (rabbit polyclonal IgG (Gene Tex)), and α-Tubulin (mouse monoclonal (Novus Biologicals)) were used. After 1-hour incubation in a horseradish peroxidase-conjugated secondary antibody, immunoreactive bands were visualized by enhanced chemiluminescence (ECL prime, GE Healthcare) and ImageQuant LAS4000 mini (GE Healthcare). Intensities of the bands of interest were quantified using Image J software.

### MRI

Isoflurane-anesthetized rats underwent MRI in a prone position. The head was kept in a fixed position during the scanning. The breathing rate was monitored throughout the experiment. MRI was performed using a 7.04 Tesla (Agilent Technologies, Palo Alto, CA, USA). T2-weighted contrast images were obtained using the following parameters: echo time = 50 ms, repetition time = 2000 ms, field of view = 2.5 × 2.5 cm^2^, matrix = 512 × 512, and slice thickness = 1 mm. To select the imaging position, proton density-weighted images were obtained using the following parameters: echo time = 11 ms, repetition time = 2000 ms, field of view = 2.5 × 2.5 cm^2^, matrix = 512 × 512, and slice thickness = 1 mm.

### Hematoxylin and eosin (HE) or Klüver-Barrera (KB) staining

HE staining was performed to observe the tissue and vascular changes according to the standard procedure. Briefly, sections were stained with Mayer’s hematoxylin for 3 min and then washed in running tap water for 10 min. Thereafter, the sections were stained with eosin for 90 s. These sections were subsequently dehydrated and cleared using alcohol and xylene, respectively. The vascular remodeling structure in HE staining was observed using microscopy (IX73, Olympus, Tokyo, Japan). We identified vascular remodeling as perivascular enlargement and vessel wall thickening [19]. The image acquisition region was randomly selected so that a cortical blood vessel was always included in the image. Ten vessels per rat, that is, 30 vessels per group, were acquired. All images within an experiment were acquired under the same microscope settings.

KB staining was performed to observe the demyelination according to the standard procedure. Briefly, sections were stained with Luxol Fast Blue solution in a 56 °C oven overnight and then washed in 95% alcohol and distilled water. Thereafter, the sections were stained with lithium carbonate solution for 30 s. Then, sections continued to undergo differentiation in 70% alcohol until the gray matter was clear and the white matter sharply defined. Next, they were counterstained with cresyl violet acetate. These sections were subsequently dehydrated and cleared using alcohol and xylene, respectively. The severity of the white matter lesions was graded as reported previously [20]. We also confirmed the presence of a white matter lesion as the formation of marked vacuoles or disappearance of myelinated fibers. We observed images of every slide by microscopy (IX73, Olympus, Tokyo, Japan).

### Statistical analysis

All statistical analyses were performed with JMP12 (SAS Institute, Cary, NC, USA). We used Student’s *t* test, Dunnett’s test, or Tukey-Kramer test. Error bars represent the means ± SEM in all figures. A *p* value of < 0.05 was considered significant.

## Results

### DOCA-salt-mediated hypertension induces abnormal parenchymal and cerebrovascular morphologies

Compared with control, placebo groups remained normotensive (Fig. 1b) and did not show any abnormal findings in histology or MR images (Additional file 1: Figure S2c and S5). DOCA2W showed a marked elevation of the blood pressure in the absence of any clinical, histopathological and MRI features of CSVD (Fig. 1b, c, Additional file 1: Figure S5 and Table 1). DOCA3W demonstrated several changes in addition to the sustained blood pressure elevation. First, DOCA3W showed clinical symptoms including hemiparesis, a decreased food intake, and bleeding from the tail vein (Table 1). Second, vascular remodeling in the cortex and the formation of vacuoles in the white matter were apparent (Fig. 1c). Third, focal high- and low-intensity areas were seen on T2-weighted on MR images (Additional file 1: Figure S5a and S5b). In DOCA4W, we observed shrinkage of the astrocyte foot processes (Additional file 1: Figure S1), a marked decrease in movement, myelin degeneration, and diffuse high-intensity areas on MR images, in addition to hypertension.

### Morphological changes of microglia precede the appearance of histopathological abnormalities

In the control and placebo groups, resting microglia, morphologically characterized by their fine processes, were observed sparsely in the cerebral parenchyma (Fig. 2a, left column). In DOCA2W, round-shaped microglia with shortened processes were observed juxtaposing vessels in the cerebral parenchyma (Fig. 2a, middle column), in the absence of histopathological abnormalities (Fig. 1c). Thereafter, morphological changes of microglia further progressed. In DOCA3W, more amoeboid microglia accumulated around structurally altered vessels (Fig. 2a, right column). In DOCA4W, amoeboid microglia were widespread across the cortex and white matter (Additional file 1: Figure S3a). The number of microglia juxtaposing vessels in the cortex was significantly increased in DOCA2W and DOCA3W (Fig. 2c).

We distinguished microglia from perivascular macrophages (PVM) by their Iba-1 intensity and morphology, based on a previous report that the expression of Iba-1 is weak in macrophages [21]. We confirmed this using a macrophage-specific marker, CD163 [22]. CD163-negative microglia showed intense Iba-1 immunoreactivity and processes. On the other hand, CD163-positive PVM showed faint Iba-1 immunoreactivity and a flattened shape (Fig. 2b). Moreover, the distribution of microglia was different from that of the PVM. CD163-positive macrophages were never observed to be accumulated in the inflammatory lesion (Additional file 1: Figure S3a).

### Activated perivascular microglia express a pro-inflammatory pattern

For characterization of the morphologically activated perivascular microglia, we first studied the expression of CD68 as a pro-inflammatory M1 marker. In the control group, microglia were CD68-negative (Fig. 3a, left column). While most of the microglia were CD68-negative in DOCA2W (Fig. 3a, middle column), CD68-positive, Iba1-positive microglia significantly increased around vessel walls and parenchyma in DOCA3W (Fig. 3a, right column). Quantitative analysis showed that the percentage of CD68-positive microglia increased significantly in DOCA3W compared with the control (Fig. 3b). As for the PVM, a sparse distribution pattern of CD68-positive PVMs was similar among the control, placebo, and DOCA groups (Fig. 3a). Biochemical analysis indicated that the expression level of inducible nitric oxide synthase (iNOS) as another pro-inflammatory M1 marker was increased in DOCA3W (Fig. 3c and d). Next, we studied the expression of CD206 as an anti-inflammatory M2 marker. Perivascular microglia did not express CD206 in rat brains of any groups (Fig. 4a), except for a few CD206-positive microglia around hemorrhage sites (Additional file 1: Figure S3b). As for the PVM, a sparse distribution pattern of CD206-positive PVMs was similar among in the control, placebo, and DOCA groups (Fig. 4a). Biochemical analysis also indicated that the expression level of arginase-1, another anti-inflammatory M2 marker, was not changed in the rat brains of any groups (Fig. 4b and c). Taken together, direct M1 activation, but not the M2 state, was identified in our DOCA-salt model.

### Activated microglia transiently expressed a cell proliferation marker, Ki-67, prior to M1 switching

The total number of microglia did not change between the control and placebo groups, whereas it significantly increased in the DOCA group (Additional file 1: Figure S4a). In the control and placebo groups, no microglia expressed Ki-67 (Fig. 5a, c and Additional file 1: Figure S4c). The rate of Ki-67-positive microglia significantly increased to 22% of the total microglia in DOCA2W, peaked to 54% in DOCA3W, and then decreased to the baseline level in DOCA4W (Fig. 5a and c). A similar increase in the number of Ki-67-positive microglia was also observed (Additional file 1: Figure S4b). The rate of Ki-67-positive perivascular microglia peaked to 55% of the total perivascular microglia in DOCA2W and remain at the same level in DOCA3W (Fig. 5d). Rates of both Ki-67-positive and M1-state microglia were highest in DOCA3W (Fig. 5e and f). In DOCA4W, proliferative M1-state microglia markedly decreased (Fig. 5c and d). In contrast, PVM did not express Ki-67 in any group (Fig. 5b).

## Discussion

**Fig. 6:**
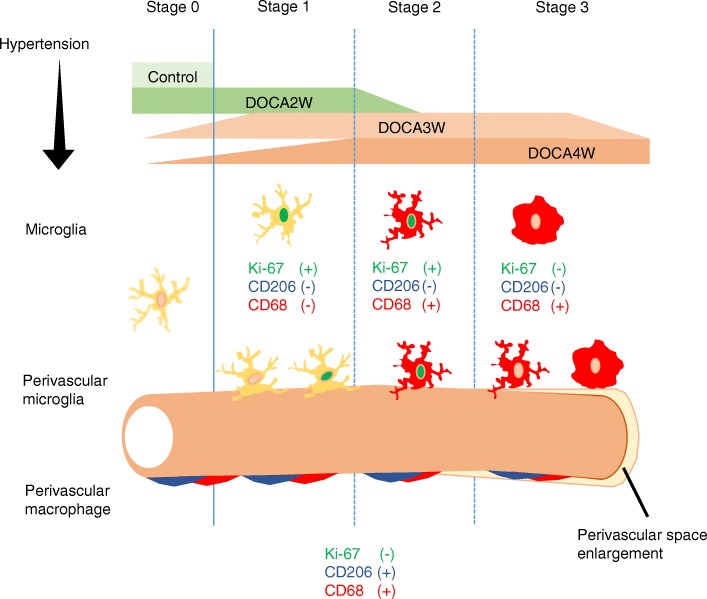
Summary of the dynamics of microglia in the process of the chronic hypertensive cerebrovascular disorder

The dynamics of microglia activation in chronic hypertension model to date have been poorly understood. In the present study, we showed microglia undergo dynamic morphological changes in the early stages of chronic hypertension using DOCA-salt-induced hypertension Wistar rats. At first, proliferative microglia juxtaposed to the cerebral vessels. Next, they switch to the pro-inflammatory M1 state, but not to the anti-inflammatory M2 state (Fig. 6). On the other hand, dynamic pathological changes of macrophages were not observed in the DOCA-salt rats. In the tMCAO model, the PVM infiltrating from vessels plays crucial roles in the enhancement of ischemic damage [23, 24]. However, this was not observed in our model (Figs. 3a and 4a and Additional file 1: Figure S3a). These results suggest that microglia, rather than PVM, are the key initial players in the process of cerebral vessel damage in the chronic hypertension model.

Another chronic hypertension model, angiotensin II (AngII)-induced hypertension model mice, showed that PVMs play important roles in neurovascular regulation [25]. Administration of AngII reaches the perivascular space and acts on AngII type 1 receptors on PVMs, which results in the activation of NADPH oxidase 2 and reactive oxygen species production. These oxidative stresses lead to neurovascular dysfunction within 2 weeks of AngII administration. However, in human, blood-AngII levels are usually normal in benign and uncomplicated essential hypertension [26]. DOCA-salt rat is characterized by low renin-AngII levels [27, 28], and BBB permeability is sustained in the early stage (at least 3 weeks after DOCA-salt administration) of hypertension in DOCA-salt rat [27, 29]. The present study aimed to evaluate the impact of the progression of chronic hypertension induced experimentally, in order to exclude from our study the potential influence of genetic factors on microglia density and/or phenotypes around cerebral vessels. We have therefore selected the Wistar DOCA-salt hypertension rat model instead of the more popular spontaneous hypertension rat (SHR) or stroke-prone spontaneous hypertension rat (SHR-SP) that carry strong differential genetic backgrounds [30, 31]. We, therefore, consider that DOCA-salt-induced hypertension Wistar rat model is suitable for the study of chronic hypertension in cerebrovascular diseases.

Microglia activation with a transient proliferative state and pro-inflammatory M1 state was observed earlier than cerebral vessel damage in our model. Regarding abnormal findings of the cerebral vasculature, perivascular space enlargement was observed in DOCA3W, and apparent BBB breakdown identified by astrocyte foot shrinkage was noted in DOCA4W (Additional file 1: Figure S1). These results are consistent with a previous study indicating that BBB permeability remained unchanged in DOCA3W mice using the Evans Blue extravasation method [27].

Regarding microglia dynamics, our findings differed from those of previous reports using acute cerebrovascular disease models, such as tMCAO. In the tMCAO model, microglia were activated to an anti-inflammatory M2 state followed by a transition to a pro-inflammatory M1 state [11]. The variation in microglia dynamics between chronic hypertension and acute ischemic models may be due to the difference of microglia-activating factors. In the tMCAO model, various factors were produced in the ischemic brain several hours or days after reperfusion. Interferon regulatory factor 4, known as an M2-switching factor, was rapidly upregulated within 2 h after ischemic insult [32]. HMGB1, one of the DAMPs, was produced several hours after ischemic events, and HMGB1 was able to activate microglia [16]. ATP or excessive glutamate was immediately released from necrotizing neuronal cells and could activate microglia [33]. In a chronic hypertension model, other than the DOCA-model, a previous study using partial renal artery occlusion model showed that microglia were activated to an inflammatory state within 5 weeks after the operation [34]. In this model, chronic hypertension increased the expression of adhesion molecules such as JAM-1, ICAM-1, and VCAM-1 on the cerebral endothelium and this led to deposition of platelets. Deposited platelets produced CD40L, which mediated the activation of pro-inflammatory microglia and activated NFκB and mitogen-activated protein kinase signaling in microglia.

A previous report showed that extracellular signal-regulated kinase (ERK)-activated microglia acquired a proliferative ability and produced mainly pro-inflammatory cytokines, which cause synaptic and neuronal losses in the brain and result in lethal neurodegenerative disease in adult mice [35]. This suggests that microglia proliferate prior to activation of the M1 state. We, therefore, analyzed the expression of Ki-67, as a cell proliferation marker, in microglia by applying immunostaining to our model. In our model, proliferative microglia had a close relationship with M1-state activation. Interestingly, such relationship was recently demonstrated using transgenic mice harboring somatic BRAF mutation, p.V600E. Activation of the MEK-ERK pathway induces microglia proliferation and is associated with an upregulation of pro-inflammatory cytokines from the proliferative microglia [35]. The rate of Ki-67-positive microglia significantly increased in DOCA2W, peaked in DOCA3W, and then decreased to the baseline in DOCA4W (Fig. 5a and c). On the other hand, the rate of Ki-67-positive perivascular microglia peaked in DOCA2W and DOCA3W (Fig. 5d) and similarly decreased to the baseline in DOCA4W. These results indicate that resting microglia were preferentially activated around the vessels, and those perivascular microglia acquired the ability to proliferate earlier than other microglia apart from the vessels. Our results suggest that specific cytokines released from the vessels induced perivascular microglia to enter a proliferative state. Further studies are required to clarify the molecular signaling involved in this phenomenon.

According to our immunohistochemical study, we concluded that perivascular microglia were from residential microglia. We distinguished microglia from macrophages by Iba-1 intensity and cell morphology. In the present study, we did not employ flow cytometry, lineage-specific markers, or other reporter methods. Flow cytometry succeeded to detect infiltration of macrophages in the ischemic brain by using tMCAO mice in which ischemic changes were observed globally in re-perfusion area [15]. However, in DOCA-salt rats, localization of vascular damages was sparse, and hence, we performed immunohistochemical analysis rather than flow cytometry analysis of brain homogenate. According to a previous study, TMEM119 antigen is expressed specifically in residential microglia in the brain but not in macrophages [36]. Unfortunately, we failed to detect TMEM119 in rat brain using antibody raised against mouse TMEM119 [37]. This may be due to the difference of amino acid sequence in the epitope region between mouse and rat. Finally, reporter method will be a powerful strategy and may be possible if the DOCA-salt model is prepared by transgenic mice expressing reporter gene such as EGFP under TMEM promoter. We like to leave this strategy for our future plan to explore the process of microglia activation under hypertension.

## Conclusions

The present study demonstrates that perivascular microglia proliferate transiently and subsequently underwent direct M1 switching, prior to cerebral vessel destruction. Our findings raise the intriguing possibility of a link between perivascular microglia activation and initiation of cerebrovascular diseases induced by chronic hypertension, and that both anti-hypertensive therapy and the fine-tuning of microglia proliferation might generate a synergistic effect.

## Additional file

Additional file 1: **Figure S1.** Morphological changes of astrocyte foot processes making up the small vessel wall in DOCA-salt rats. **Figure S2.** Quantitative analysis of vessel wall thickening and perivascular space enlargement. **Figure S3.** Distribution of perivascular macrophages and microglia in DOCA4W, and presence of CD206-positive M2-state microglia around a site of hemorrhage. **Figure S4.** Quantitative analysis of microglia. **Figure S5.** Sequential MRI analysis of rat brains. (PPTX 8857 kb)

## Abbreviations

AngII: Angiotensin II
BBB: Blood-brain barrier
CSVD: Cerebral small vessel disease
DAMPs: Damage-associated molecular patterns
DAPI: 4′, 6-Diamino-2-phenylindole
DOCA: Deoxycorticosterone acetate
ERK: Extracellular signal-regulated kinase
GFAP: Glial fibrillary acidic protein
HE: Hematoxylin and eosin
HMGB1: High-mobility group box 1
Iba-1: Ionized calcium-binding adapter molecule 1
iNOS: Inducible nitric oxide synthase
KB: Klüver Barrera
MRI: Magnetic resonance imaging
PBST: Phosphate-buffered saline containing 0.1% Tween 20
PVMs: Perivascular macrophages
tMCAO: Transient middle cerebral artery occlusion
