# Supplementary material for: Transiently proliferating perivascular microglia harbor M1 type and precede cerebrovascular changes in a chronic hypertension model
Source: J Neuroinflammation. 2019 Apr 10;16:79. doi: 10.1186/s12974-019-1467-7 (PMC6456949; doi:10.1186/s12974-019-1467-7)
Supplement: Supplementary file 1 — Figure S1. Morphological changes of astrocyte foot processes making up the small vessel wall in DOCA-salt rats. Figure S2. Quantitative analysis of vessel wall thickening and perivascular space enlargement. Figure S3. Distribution of perivascular macrophages and microglia in DOCA4W, and presence of CD206-positive M2-state microglia around a site of hemorrhage. Figure S4. Quantitative analysis of microglia. Figure S5. Sequential MRI analysis of rat brains. (PPTX 8857 kb) [file 12974_2019_1467_MOESM1_ESM.pptx]

## Slide 1
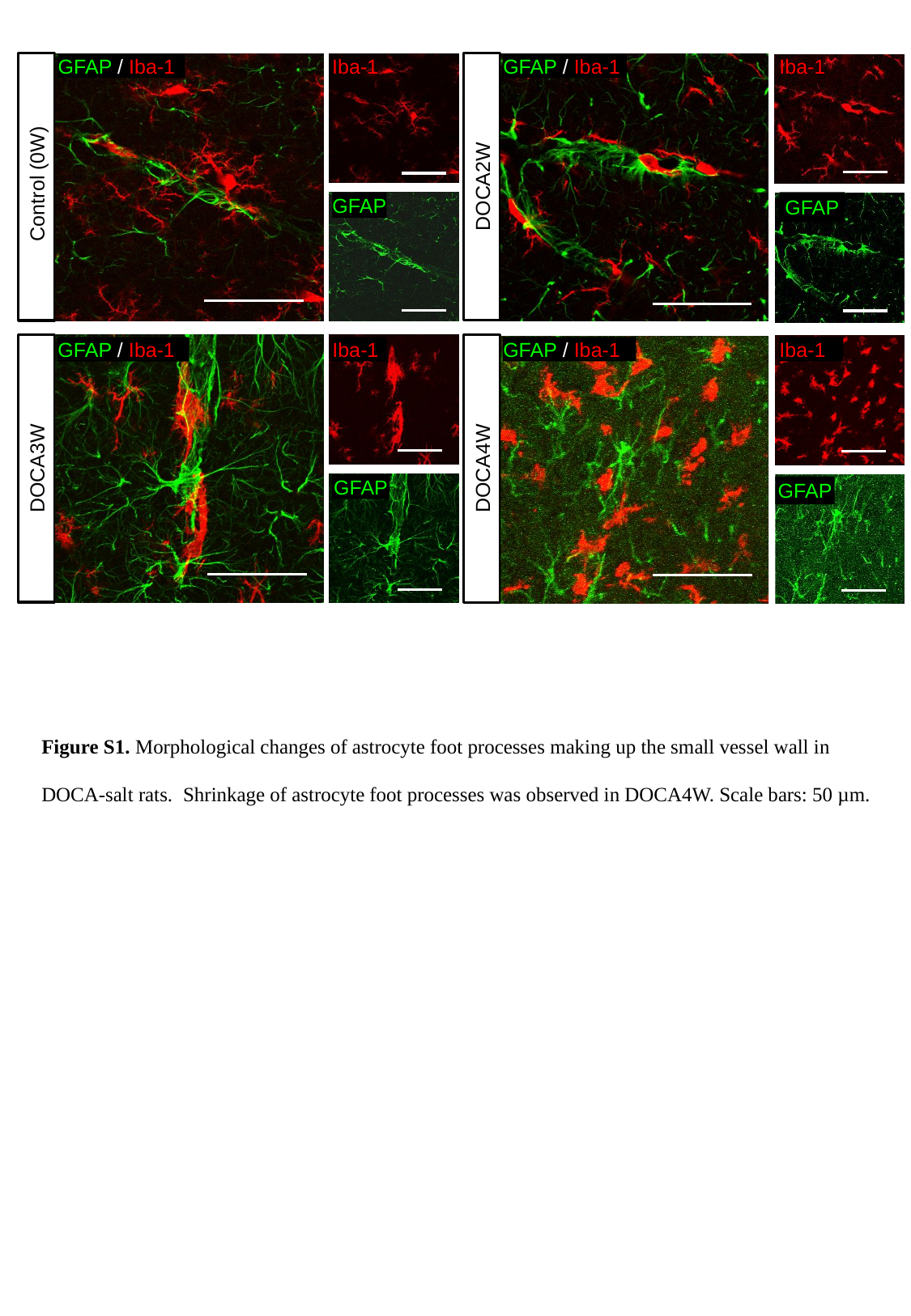

GFAP / Iba-1
GFAP / Iba-1
Iba-1
Iba-1
DOCA2W
 Control (0W)
GFAP
GFAP
GFAP / Iba-1
Iba-1
GFAP / Iba-1
Iba-1
20μm
DOCA3W
DOCA4W
GFAP
GFAP
20μm
Figure S1. Morphological changes of astrocyte foot processes making up the small vessel wall in DOCA-salt rats. Shrinkage of astrocyte foot processes was observed in DOCA4W. Scale bars: 50 µm.
20μm
20μm

## Slide 2
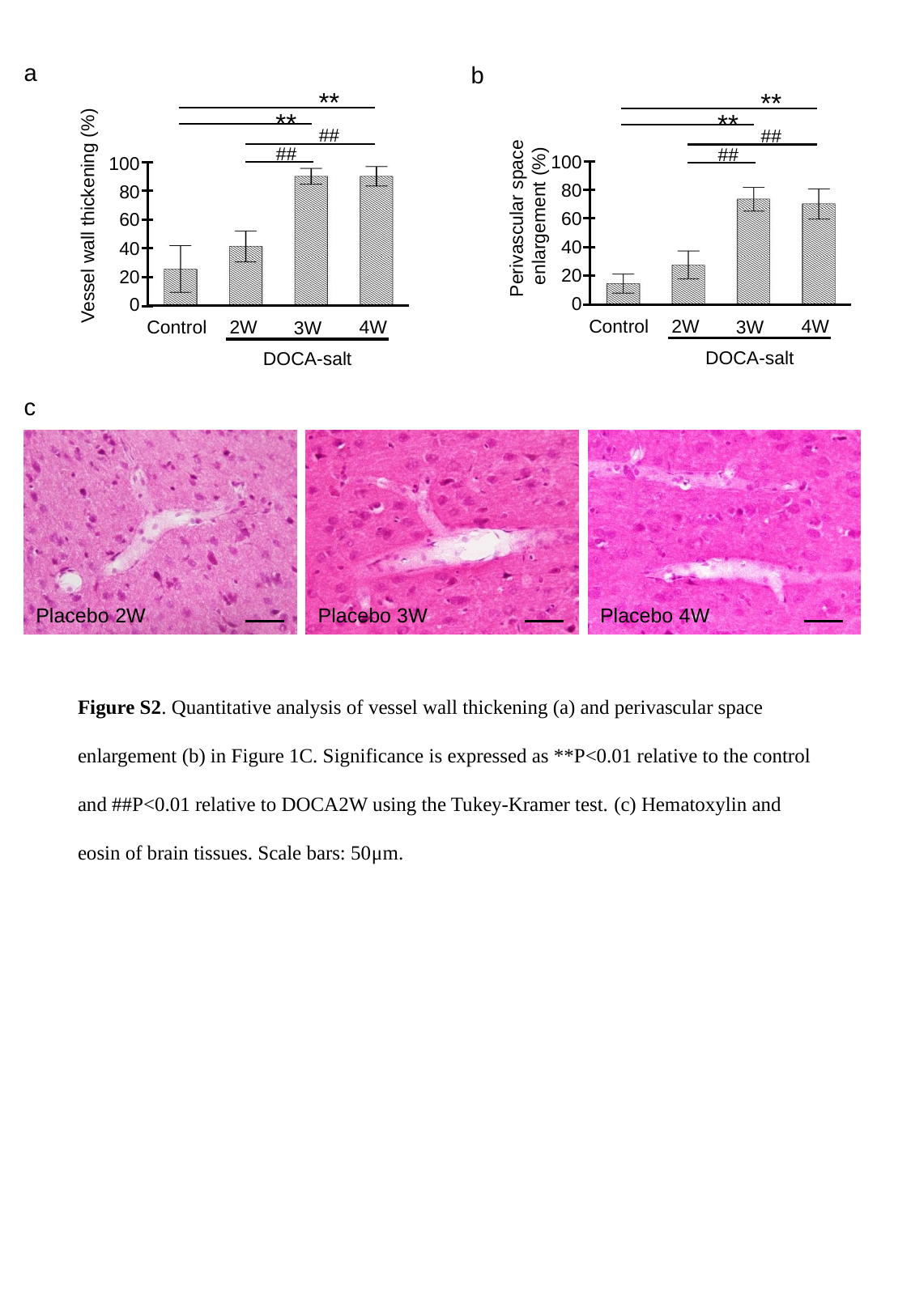

a
b
**
**
##
##
**
**
##
##
100
80
60
40
20
0
Control
2W
4W
3W
DOCA-salt
100
80
60
40
20
0
Control
2W
4W
3W
DOCA-salt
Perivascular space
enlargement (%)
Vessel wall thickening (%)
c
Placebo 3W
Placebo 4W
Placebo 2W
Figure S2. Quantitative analysis of vessel wall thickening (a) and perivascular space enlargement (b) in Figure 1C. Significance is expressed as **P<0.01 relative to the control and ##P<0.01 relative to DOCA2W using the Tukey-Kramer test. (c) Hematoxylin and eosin of brain tissues. Scale bars: 50μm.

## Slide 3
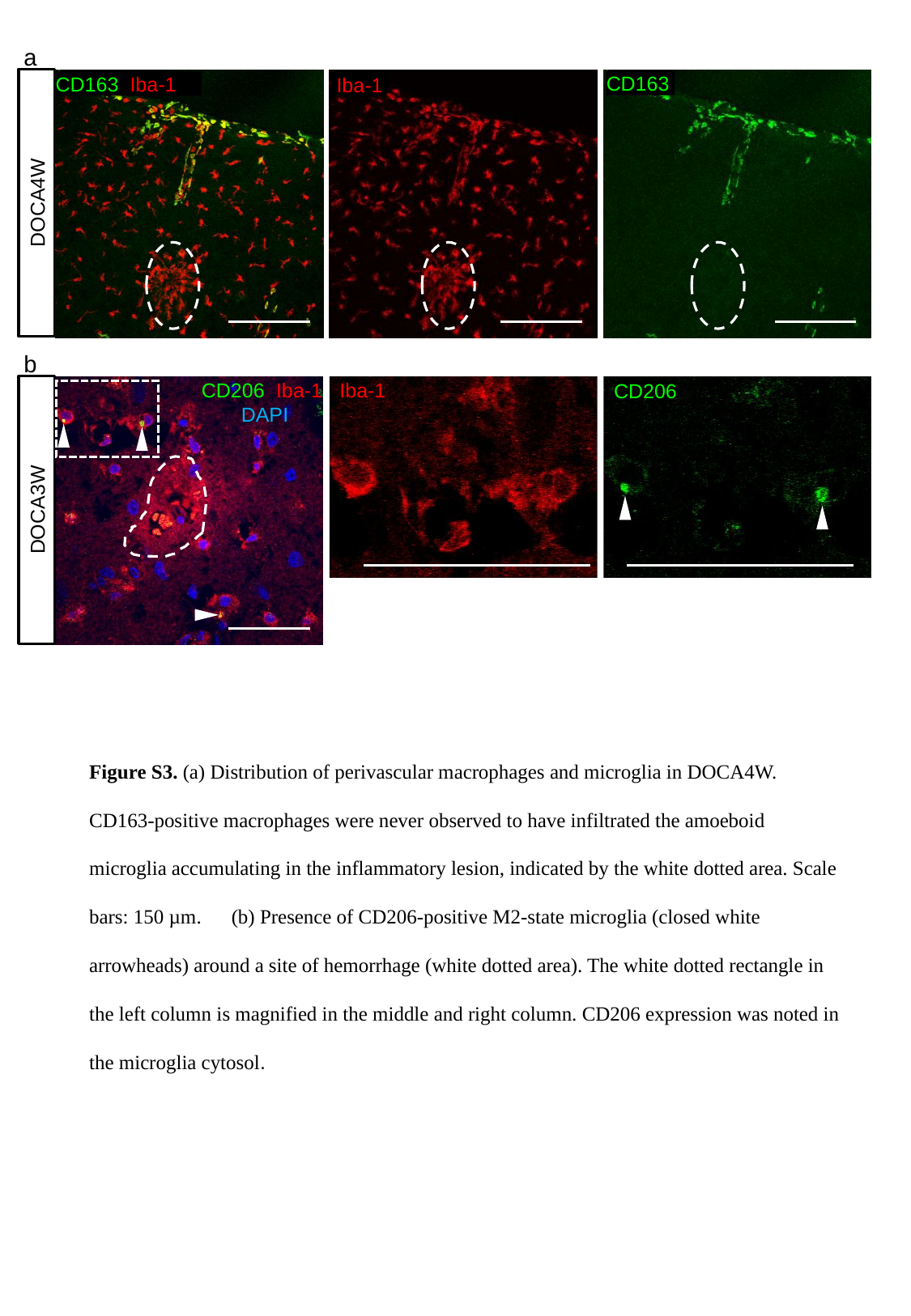

a
CD163
CD163 Iba-1
 Iba-1
DOCA4W
b
Iba-1
CD206
CD206 Iba-1 DAPI
DOCA3W
Figure S3. (a) Distribution of perivascular macrophages and microglia in DOCA4W. CD163-positive macrophages were never observed to have infiltrated the amoeboid microglia accumulating in the inflammatory lesion, indicated by the white dotted area. Scale bars: 150 µm.　(b) Presence of CD206-positive M2-state microglia (closed white arrowheads) around a site of hemorrhage (white dotted area). The white dotted rectangle in the left column is magnified in the middle and right column. CD206 expression was noted in the microglia cytosol.

## Slide 4
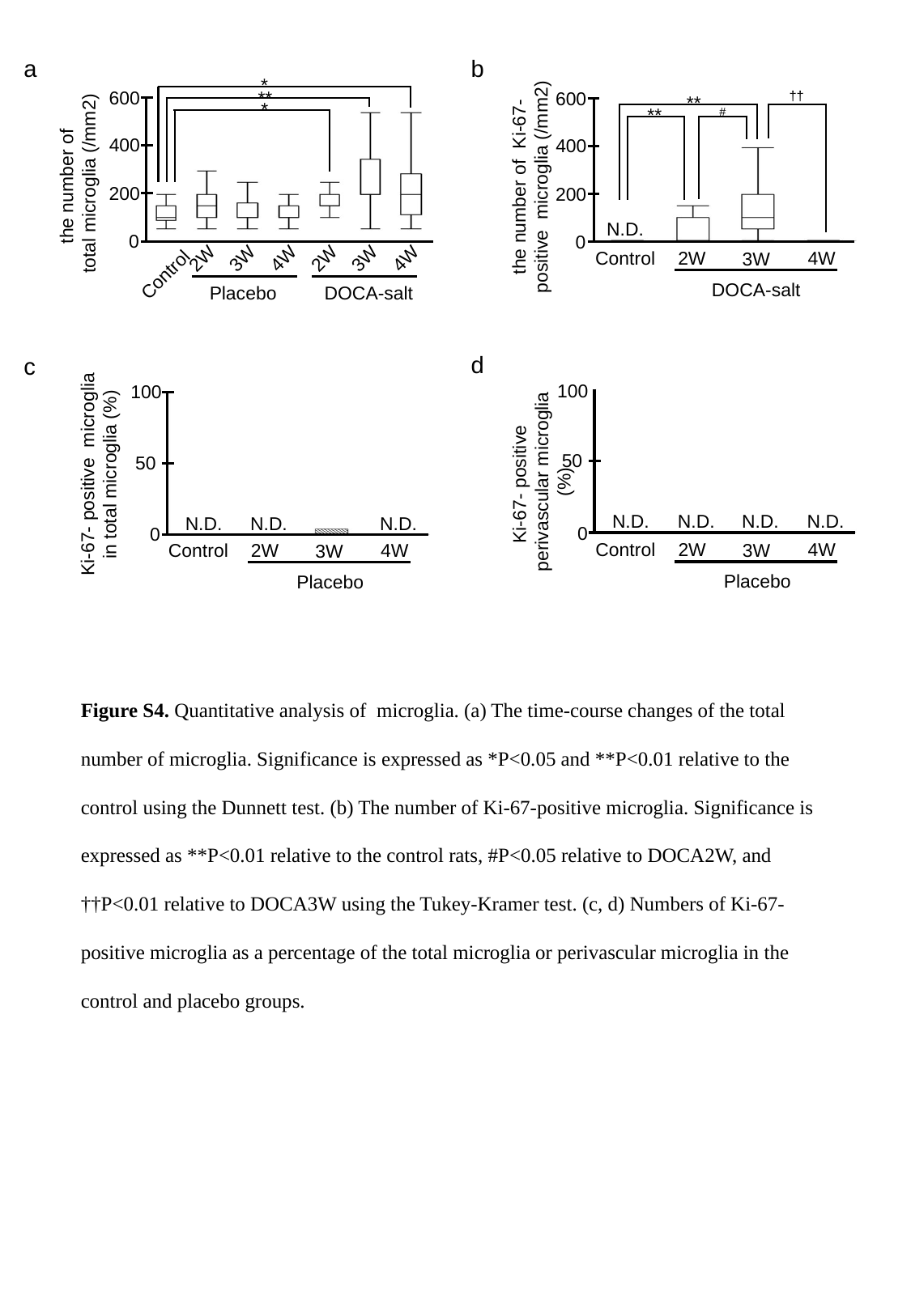

a
b
*
600
400
200
 0
**
600
400
200
 0
††
**
*
**
#
the number of
 total microglia (/mm2)
the number of Ki-67-positive microglia (/mm2)
N.D.
3W
3W
2W
4W
2W
4W
Control
2W
4W
3W
DOCA-salt
Control
Placebo
DOCA-salt
d
c
100
100
50
50
Ki-67- positive microglia
in total microglia (%)
Ki-67- positive
perivascular microglia (%)
N.D.
N.D.
N.D.
N.D.
N.D.
N.D.
N.D.
 0
 0
Control
2W
4W
Control
2W
4W
3W
3W
Placebo
Placebo
Figure S4. Quantitative analysis of microglia. (a) The time-course changes of the total number of microglia. Significance is expressed as *P<0.05 and **P<0.01 relative to the control using the Dunnett test. (b) The number of Ki-67-positive microglia. Significance is expressed as **P<0.01 relative to the control rats, #P<0.05 relative to DOCA2W, and ††P<0.01 relative to DOCA3W using the Tukey-Kramer test. (c, d) Numbers of Ki-67-positive microglia as a percentage of the total microglia or perivascular microglia in the control and placebo groups.

## Slide 5
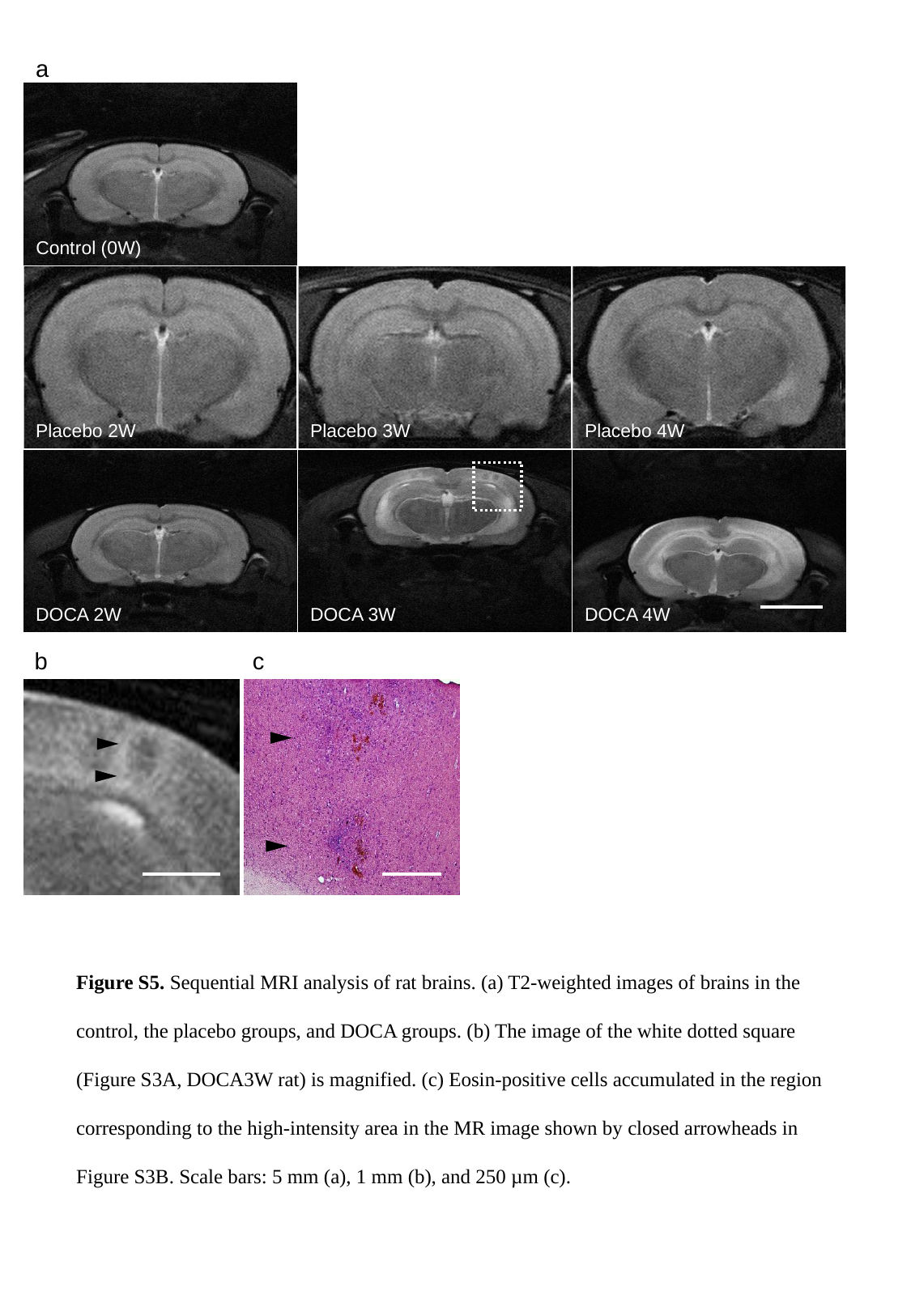

a
Control (0W)
Placebo 2W
Placebo 3W
Placebo 4W
DOCA 3W
DOCA 4W
DOCA 2W
b
c
Figure S5. Sequential MRI analysis of rat brains. (a) T2-weighted images of brains in the control, the placebo groups, and DOCA groups. (b) The image of the white dotted square (Figure S3A, DOCA3W rat) is magnified. (c) Eosin-positive cells accumulated in the region corresponding to the high-intensity area in the MR image shown by closed arrowheads in Figure S3B. Scale bars: 5 mm (a), 1 mm (b), and 250 µm (c).
